# Moderate intensity continuous training mitigates hypertension-induced renal fibrosis by inhibiting HIF-1α-mediated autophagy

**DOI:** 10.3389/fphys.2025.1529811

**Published:** 2025-03-27

**Authors:** Yun Li, Xinyu Yang, Zhuo Chen, Wenyu Dong, Xinhua Chen, Wenhao Wang, Lingang Li, Wenjun Ma, Qing Chang

**Affiliations:** ^1^ The Affiliated Rehabilitation Hospital, Chongqing Medical University, Chongqing, China; ^2^ Department of Orthopedic Surgery, The First Affiliated Hospital of Chongqing Medical University, Chongqing, China; ^3^ Department of Rehabilitation Medicine, The First Affiliated Hospital of Chongqing Medical University, Chongqing, China; ^4^ The College of Exercise Medicine, Chongqing Medical University, Chongqing, China

**Keywords:** moderate intensity continuous training, HIF-1α, autophagy, hypertensive renal fibrosis, high-intensity interval training

## Abstract

**Introduction:** Hypertension is a significant risk factor for kidney disease. Aerobic exercise has demonstrated positive effects in managing hypertensive nephropathy. However, the impact of exercise on hypertensive nephropathy remains contentious due to variations in exercise protocols. This study aimed to compare the effects of moderate-intensity continuous training (MICT) and high-intensity interval training (HIIT) on renal fibrosis in spontaneously hypertensive rats (SHRs).

**Methods:** SHRs underwent a 10-week treadmill training with moderate-intensity continuous training (MICT) and high-intensity interval training (HIIT). The blood pressure in rats was measured following the conclusion of the final exercise training session. The renal function, levels of HIF-1α, fibrosis, and autophagy were evaluated by immunostaining and western blot in rat kidneys. The AKT/mTOR signaling pathway was also investigated. In vitro, we also treated angiotensin II-induced HK-2 cells with inhibited or overexpressed HIF-1α and tested the changes in fibrosis and autophagy by immunostaining and western blot. Following treatment with lysosomal inhibitors (chloroquine), the expression of fibrosis was further investigated.

**Results:** Our findings indicated that MICT improved renal function and inhibited fibrosis through downregulation of HIF-1α and autophagy, whereas HIIT did not lead to significant improvement. Additionally, inhibition of HIF-1α attenuates Ang II-induced fibrosis and autophagy in HK-2 cells. HIF-1α overexpression had the opposite effect. CQ further alleviates fibrosis.

**Conclusion:** These findings had elucidated the potential of MICT to ameliorate renal fibrosis caused by hypertension by targeting HIF-1α-regulated autophagy.

## 1 Introduction

A major risk factor for diabetes, heart disease, and kidney disease, hypertension is a global health concern with significant economic implications (Burnier and Damianaki, 2023; Burnier and Egan, 2019; Li et al., 2024). Hypertensive nephropathy (HN) is characterized by persistent hypertension, which leads to arteriolar lesions within the kidneys, ischemic injury, glomerulosclerosis, and interstitial fibrosis, ultimately progressing to end-stage renal disease (Gupta et al., 2023; Hao et al., 2024; Mennuni et al., 2014). Persistent hypertension also damages renal tubular cells, further contributing to the development of renal interstitial fibrosis (Wu et al., 2023). In this condition, tubular atrophy and extracellular matrix deposition occur, resulting in the production of cytokines that activate interstitial myofibroblasts (Humphreys, 2018; Li et al., 2022). The mechanisms underlying these tubular changes are highly complex, with hypoxia recognized as a critical environmental factor. The hypoxia response is a key mechanism by which the kidneys adapt to pathological hypoxic conditions. Due to their constant state of active metabolism, the kidneys require a substantial oxygen supply (Natarajan et al., 2021; Tian and Liang, 2021). Several studies have reported that spontaneously hypertensive rats (SHR) exhibit greater renal cortical hypoxia compared to normotensive Wistar Kyoto rats (WKY), characterized by increased oxygen consumption (Natarajan et al., 2021; Tian and Liang, 2021). Hypoxia is linked to both acute and chronic kidney diseases and is a contributing factor to renal interstitial fibrosis (Liu et al., 2017).

Preventing hypoxic injury is a critical therapeutic strategy in managing HN (Luo et al., 2015; Natarajan et al., 2021). Hypoxia-inducible factor-1α (HIF-1α) plays a key regulatory role under mammalian hypoxic conditions. The knockout or inhibition of HIF-1α has been shown to effectively alleviate hypertensive renal injury (Huang et al., 2019a). In unilateral ureteral obstruction (UUO), the expression of associated proteins, such as microtubule-associated protein light chain 3 (LC3), Beclin1, HIF-1α, and α-smooth muscle actin (α-SMA), has been upregulated (Kim et al., 2021). Studies have shown that HIF-1α overexpression acts as a pathogenic factor in the development of renal interstitial fibrosis and promotes disease progression (Livingston et al., 2023). The elevated HIF-1α levels in the kidney fibrosis model could further drive autophagy and renal fibrosis (Luo et al., 2015; Natarajan et al., 2021). Therefore, it is hypothesized that in the context of hypertensive renal injury, HIF-1α mediates the sustained activation of autophagy.

Exercise aids in managing blood pressure and ameliorates hypertensive nephropathy by reducing proteinuria and inhibiting fibrosis (Dong et al., 2024). Recent studies suggest that chronic hypertension increases the susceptibility of kidney tissue susceptibility to fibrosis, oxidative stress, and inflammation, which ultimately results in renal damage; conversely, exercise can reverse these detrimental effects, thereby contributing to kidney protection (Dong et al., 2024; Huang C. et al., 2018; Yamakoshi et al., 2022). However, the impact of physical exercise on kidney function may be contradictory, depending on the intensity of physical activity and the extent of renal impairment. Variations in exercise intensity might affect hypertensive nephropathy differently. Recent studies suggest that HIIT may be more effective than MICT in reducing diastolic blood pressure and improving outcomes in hypertensive metabolic syndrome (Jo et al., 2020; Leal et al., 2020). However, reliable evidence regarding the physiological effects of HIIT on kidney function remains limited. Individuals with pre-existing conditions may experience secondary renal injury from excessively intense training, potentially negating the physiological benefits gained from exercise (Kodikara et al., 2023; Mizokami et al., 2024). The effects of MICT and HIIT on hypertensive nephropathy continue to be a topic of ongoing debate.

In this study, we utilized MICT and HIIT to explore the effects of exercise modalities on renal HIF-1α protein levels and renal fibrosis in SHRs. Autophagy and the AKT/mTOR signaling pathway were also evaluated. In addition, we inhibited or overexpressed HIF-1α in HK-2 cells to identify the underlying molecular mechanisms by which exercise ameliorates hypertensive nephropathy.

## 2 Materials and methods

### 2.1 Animals

All rats in the project were housed under standard pathogen-free conditions with a controlled temper ature (22°C ± 2°C) and a 12/12 h light/dark cycle. The subjects were allowed *ad libitum* access to the same standard diet and water. Animal experiments were conducted in accordance with the National Animal Protection and Use Guidelines and were approved by the Animal Ethics Committee of Chongqing Medical University (IACUC-CQMU). Eighteen healthy male spontaneously hypertensive rats (SHRs), aged 8 weeks, and age-matched set of six Wistar-Kyoto (WKY) rats were purchased from SiPeiFu Laboratory (Beijing, China). The rats were randomly allocated to four groups (n = 24): 1) WKY sedentary (WKY-S) group, 2) SHR sedentary (SHR-S) group, 3) SHR moderate-intensity continuous training (SHR-M) group, and 4) SHR high-intensity interval training (SHR-H) group.

### 2.2 Exercise training protocol

The rats were exercised on a specialized treadmill (SoftLong Technology, Shanghai, China) connected to software for continuous speed monitoring. All rats underwent a 7-day adaptation training (30 min/day at 8 m per minute), followed by an incremental exercise test (IET) to determine the maximum running speed (Smax). The rats were placed on the treadmill at an initial speed of 8 m/min with a 0° incline, with the speed increasing by 2 m/min every 2 min until exhaustion. The speed at this stage was defined as Smax. The treadmill exercise followed protocols described in previous studies (Luo et al., 2023; Poole et al., 2020; Tucker et al., 2015). For SHR-M group, speeds were set at 40%–50% (16–18 m/min) of Smax. Each MICT session lasted 60 min. For the SHR-H group, the rats had been subjected to a regimen comprising 10 high-intensity intervals repetitions, 2-min stationary period followed by 1-min sprint period at 70%–80% (28–30 m/min) of Smax. The total exercise volume was carefully matched between the two groups to ensure comparability. All training sessions were conducted 5 days per week for 10 weeks. After the exercise training of 10 weeks, the rats were maintained in the metabolic cages for 24 h to collect 24-h urine for quantitative urinary protein testing. Serum and kidney were kept at −80°C for future research.

### 2.3 Measurements of weight, heart rate and blood pressure

The weight of rats was measured using an animal scale every week. Blood pressure and heart rate of rats were assessed pre-exercise and post-exercise using the tail cuff sphygmomanometer system (BP2010-A; Softron Beijing Biotechnology Co., Ltd.).

### 2.4 Serum and urine biochemical analysis

Blood urea nitrogen (BUN), Serum creatinine (S-cr), β-N-acetyl-glucosaminidase (NAG), and 24-h urinary protein (24 h UPro) levels were measured using assay kits (Nanjing Jiancheng Bioengineering Institute, Nanjing, China) according to the manufacturer’s instructions.

### 2.5 H&E and masson staining

The kidney tissues were fixed in 4% paraformaldehyde for 24 h, progressively dehydrated, and embedded in paraffin wax blocks. The blocks were labeled according to the different treatment groups. The paraffin blocks were then sectioned, dewaxed, and washed. Hematoxylin and eosin staining were performed sequentially. Kidney tissue sections were stained using a Masson staining kit (Solarbio, Beijing, China). The samples were then observed and analyzed using a light microscope (Olympus, Japan).

Blinded histological analysis assessed tubular injury, dilatation, intra-luminal casts, and brush border loss. Fifteen fields per sample were examined at ×200 magnification. Lesions were scored as follows: 0 (normal); 1 (mild, 0%–10% involvement); 2 (moderate, 11%–25%); 3 (severe, 26%–49%); 4 (highly severe, 50%–75%); or five (extensive, >75%). The histological scores were subjected to statistical analysis.

### 2.6 Cell culture

Human kidney proximal tubule epithelial cells (HK-2) were purchased from Procell Life Science & Technology Co., Ltd (Wuhan, China). The cells were grown at 37°C in an incubator with 5% CO2 (Thermo Fisher, United States) using DMEM/F12 medium (Gibco, United States), supplemented with 10% FBS (Gibco, United States of) and 1% penicillin-streptomycin (Beyotime, Shanghai, China). HK-2 cells were incubated with various concentrations of KC7F2 (0.5, 1, or 2 μM) (MedChemExpress, Shanghai, China), a HIF-1α inhibitor, both in the absence and presence of angiotensin II (Ang II, MedChemExpress, Shanghai, China). CQ (10 μM; an autophagy inhibitor; MedChemExpress, Shanghai, China) was co-incubated with angiotensin II and KC7F2.

### 2.7 Western blotting

Renal tissues from all six rats in each group (n = 6 biologically independent experiments) or HK-2 cells were homogenized in RIPA buffer and centrifuged at 4°C and 7,490 × g for 15 min. The supernatant were denatured at 100°C for 10 min with loading buffer, and 20 μg of protein was separated by SDS-PAGE and transferred onto a nitrocellulose membrane. The membrane was blocked with 5% non-fat milk solution at room temperature for 2 h and then incubated overnight at 4°C with primary antibodies. The following dilutions were used for different primary antibodies (Table 1). The membrane was then incubated with HRP-conjugated secondary antibodies at room temperature for 1 h. Bands were detected using a chemiluminescent detection reagent (MCE, Shanghai, China). The band intensities were analyzed with ImageJ, and the target protein levels were calculated as the ratio of their grayscale values to the internal control protein (β-actin). Western blot results are derived from three independent experiments.

**TABLE 1:** List of primary antibodies used for Western blotting technique.

Target proteins	Dilution ratio	Primary antibody
Col1a1	1:1000	Zenbio,343,277
HIF-1α	1:1000	Zenbio,340,462
Vimentin	1:20,000	HUABIO, ET1610-39
LC3	1:2000	Abmart, T55992
P62	1:5000	Abmart, T55546
ATG5	1:2000	Abmart, T55766
AKT	1:5000	HUABIO, ET1609-51
p-AKT	1:5000	HUABIO, ET1607-73
mTOR	1:1000	Cell Signaling Technology, 2972S
p-mTOR	1:1000	Cell Signaling Technology, 2971S
β-actin	1:100,000	HUABIO, HA722023

### 2.8 Immunostaining analysis

As previously described, the samples were subjected to immunohistochemical and immunofluorescence staining. The antibodies and reagents employed included: anti-HIF-1α antibody (1:200, 340,462, Zenbio), anti-LC3 antibody (1:200, 14600-1-AP, Proteintech), anti-P62 antibody (1:200, T55546, Abmart), anti-Vimentin antibody (1:200, ET1610-39, HUABIO), Multi-rAb CoraLite^®^ Plus 488 Goat Anti-Rabbit Recombinant Secondary Antibody (H + L) (1:400, RGAR002, Proteintech), and Multi-rAb CoraLite^®^ Plus 594 Goat Anti-Mouse Recombinant Secondary Antibody (H + L) (1:400, RGAM004, Proteintech). Images were collected with a confocal microscope (LSM880, Carl Zeiss, Germany) or an optical microscope (BX53, Olympus, Japan) and were subsequently analyzed with ImageJ software. In rats, three random fields of view were selected per rat, and three randomly selected rats were analyzed per group. For cell experiments, three random fields of view were selected, and the analysis was performed on three independent experiments.

### 2.9 CCK8 assay

The HK-2 cells were collected and diluted to a concentration of 5 × 10^3^ cells/mL to prepare a cell suspension. This suspension was then plated in 96-well plates. The cells were exposed with angiotensin II (Ang-II; MedChemExpress, Shanghai, China) at concentrations of 1, 2.5, 5, 10, and 20 μM for 24 h. Following treatment, the medium was removed, and the Cell Counting Kit-8 (CCK-8) assay (Beyotime, Shanghai, China) was performed to each well. Following a 1-h incubation at 37°C, the absorbance was recorded at 450 nm using a microplate reader (Thermo Scientific, United States).

### 2.10 Lentivirus infection

HIF-1α-overexpression was generated by OBiO (Shanghai, China). Over-expression of HIF-1α were transfected into HK-2 cells. After 72 h, fluorescence was observed through an inverted fluorescence microscope to assess infection efficiency. The stable cell lines were selected using 1 μg/mL puromycin (Beyotime, Beijing, China) in medium. Finally, total protein was collected, and the efficiency of HIF-1α overexpression was verified by RT-qPCR and Western blotting. Additionally, lentivirus with a negative control short hairpin RNA was used to account for any effects of the lentivirus itself on transfection.

### 2.11 RNA extraction and RT-qPCR

Total RNA was extracted from cultured cells via the EZ-10 Total RNA Mini-Preps Kit (Sangon Biotech, China). For cDNA synthesis, RNA was reverse transcribed through the AMV First Strand cDNA Synthesis Kit (Sangon Biotech, China). RT-qPCR was performed with 2× SYBR qPCR Mix (Sangon Biotech, China). HIF-1α was amplified with the following primers: 5′-GGTCTAGGAAACTCAAAACCTGA-3′ and 5′-TGGCTGCATCTCGAGACTTT-3′, while β-actin was amplified with 5′-CCTGGCACCCAGCACAAT-3′ and 5′-GGGCCGGACTCGTCATAC-3′.

### 2.12 Statistical analysis

Data are expressed as means ± standard error of the mean (SEM). For comparisons between two groups, an unpaired Student’s t-test was used, while multiple group comparisons were analyzed by one-way ANOVA followed by Tukey’s *post hoc* test. All analyses were performed by the GraphPad Prism 9.5.1 software. The difference was considered statistically significant if p < 0.05.

## 3 Results

### 3.1 Effects of MICT and HIIT on body weight, blood pressure, heart rate

**FIGURE 1:**
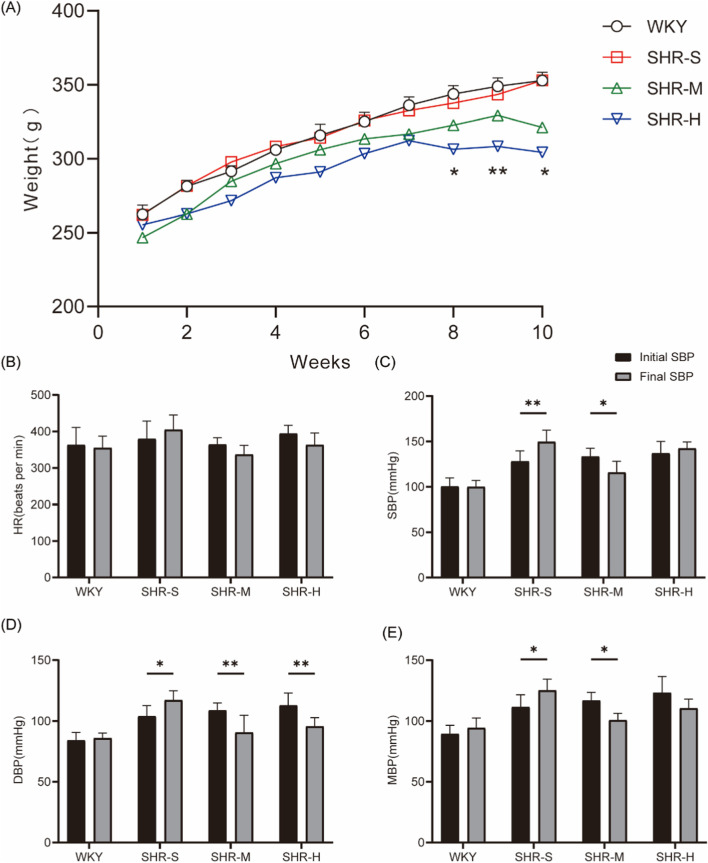
Effects of MICT and HIIT on body weight, blood pressure, heart rate **(A)** The weight of rats was measured every week. **(B)** Heart rate **(C)** Systolic blood pressure, **(D)** Diastolic blood pressure, **(E)** Mean arterial pressure and were measured before and after the exercise intervention.

First, body weights were monitored weekly throughout the training period, revealing differential weight changes among the groups (Figure 1A). Notably, a statistically significant divergence in body weight (P < 0.05) was observed between the SHR-M and SHR-H groups from weeks 8–10. After 10 weeks of exercise training, the SBP, DBP, and MBP were lower in the SHR-M group compared to before the start of exercise. Additionally, DBP decreased after exercise training in the SHR-H group, but there was no significant change in SBP and MAP. There was also no significant change in HR before and after exercise (Figures 1B–E).

### 3.2 Effects of training on hypertension-induced renal pathology

**FIGURE 2:**
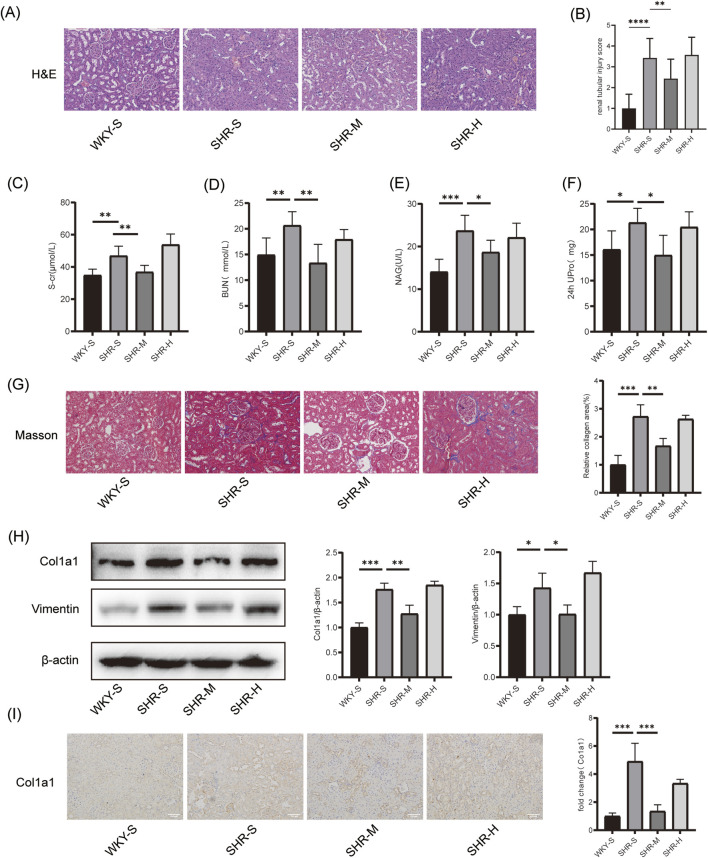
Effects of training on hypertension-induced renal pathology`renal function and fibrosis **(A)** H&E in kidney tissues. Scale bar, 100 μm. **(B)** renal tubular injury score **(C)** Serum creatinine (S-cr) **(D)** Blood urea nitrogen (BUN) **(E)** β-N-acetyl-glucosaminidase (NAG), and **(F)** 24-h urinary protein (24 h UPro) were measured to assess renal function in SHR **(G)** Masson staining in kidney tissues. Scale bar, 100 μm. **(H)** The Col1a1 and Vimentin protein expression levels in kidney tissues were measured by Western blotting. **(I)** Immunohistochemical staining of Col1a1 in kidney tissues. Scale bar, 100 μm *p < 0.05, **p < 0.01, ***p < 0.001.

The H&E staining revealed that rats in the SHR-S group exhibited marked pathological alterations in the kidney, such as altered glomerular morphology, swelling of tubular epithelial cells, and thickening of some vascular walls. These pathological alterations were mitigated in the SHR-M group, while no notable improvement was observed in the SHR-H group (Figure 2A). The tubular injury was scored by investigators blinded to the group assignments. Statistical analysis revealed significantly higher scores in the SHR-S group compared to the WKY-S group. Notably, the SHR-M group demonstrated a marked reduction in injury scores relative to the SHR-S group, whereas no statistically significant difference was observed between the SHR-H and SHR-S groups (Figure 2B).

### 3.3 Effects of training on hypertension-induced renal function and fibrosis

We assessed the histopathology and kidney function of rats after 10 weeks of different exercise interventions. Regarding kidney function, significant differences were observed between the WKY-S and SHR-S group in S-cr, BUN, NAG, and 24-h urinary protein levels. SHR-M group led to significant reductions in these indicators, whereas no significant reductions were observed in the SHR-H group (Figures 2C–F). Furthermore, the Masson staining results revealed that the fibrotic area of the renal cortices in the SHR-S group was considerably larger than in the WKY-S group. The fibrotic area was markedly reduced in the SHR-M group relative to the SHR-S group, whereas no significant differences were detected between the SHR-S and SHR-H group (Figure 2G). Western blotting analysis demonstrated that, compared to the SHR-S group, SHR-M group downregulated the expression of fibrosis markers such as Col1a1 and Vimentin proteins, while no significant differences were detected between the SHR-H and SHR-S group (Figure 2H). Similar results were observed in immunohistochemistry analysis (Figure 2I).

### 3.4 Effects of training on renal autophagy in SHRs

**FIGURE 3:**
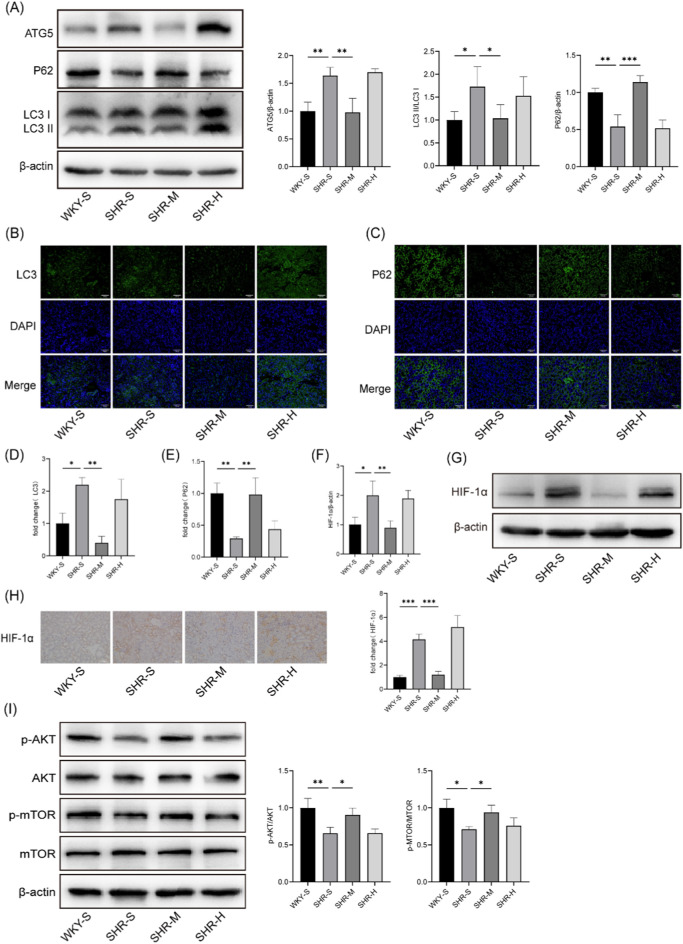
Effects of training on renal the levels of autophagy, HIF-1α and AKT/mTOR pathway in SHRs **(A)** The ATG5、P62 and LC3B protein expression levels in kidney tissues were measured by Western blotting. **(B)** Immunofluorescence staining of LC3B in kidney **(C)** Immunofluorescence staining against P62 of kidney. **(D)** Statistical chart of LC3B fluorescence intensity **(E)** Statistical chart of P62 fluorescence intensity. **(F)** Quantitative densitometry analysis of HIF-1α **(G)** Western blotting was performed to measure HIF-1α level in kidney. **(H)** The representative images and statistical graph of IHC staining for HIF-1α in the kidney tissue. Scale bar, 100 μm. **(I)** Western blotting images and quantitative densitometry analysis of p-AKT, AKT, p-mTOR, and mTOR. *p < 0.05, **p < 0.01, ***p < 0.001.

Excessive activation of autophagy plays a crucial role in the pathogenesis of renal fibrosis. Western blotting analysis revealed that, relative to the WKY-S group, the autophagy markers ATG5 and LC3B-II were significantly upregulated in the SHR-S group, while the selective autophagy substrate receptor p62 was substantially downregulated. Importantly, only SHR-M group successfully reversed these changes (Figure 3A). Likewise, immunofluorescence and immunohistochemistry provided further support for the Western blot results, showing that increased ATG5 and LC3B-II correspond to enhanced autophagosome formation, while decreased p62 reflects diminished autophagosome degradation (Figures 3B–E).

### 3.5 Effects of training on HIF-1α expression in the kidneys of SHRs

Western blotting and immunohistochemistry techniques were employed to determine the effect of different exercise regimens on HIF-1α expression in SHR kidneys (Figures 3F–H). The results indicated an increase in HIF-1α expression in the SHR-S group compared to the WKY-S group. This increase was mitigated in SHR-M, whereas SHR-H did not induce significant changes.

### 3.6 Effects of training on hypertensive nephropathy via the AKT/mTOR signaling pathway

To investigate the role of exercise in regulating hypertensive kidney injury via the AKT/mTOR signaling pathway, we examined p-AKT, AKT, p-mTOR, and mTOR protein levels. Western blotting analysis demonstrated that the p-AKT/AKT and p-mTOR/mTOR ratios were decreased in the SHR-S group compared to the WKY-S group. SHR-M increased these ratios and activated the AKT/mTOR signaling pathway in comparison to the SHR-S group. However, no significant differences were observed between the SHR-H and SHR-S group (Figure 3I).

### 3.7 Inhibition of HIF-1α ameliorated AngⅡ-induced autophagy and fibrosis in HK-2 cells

**FIGURE 4:**
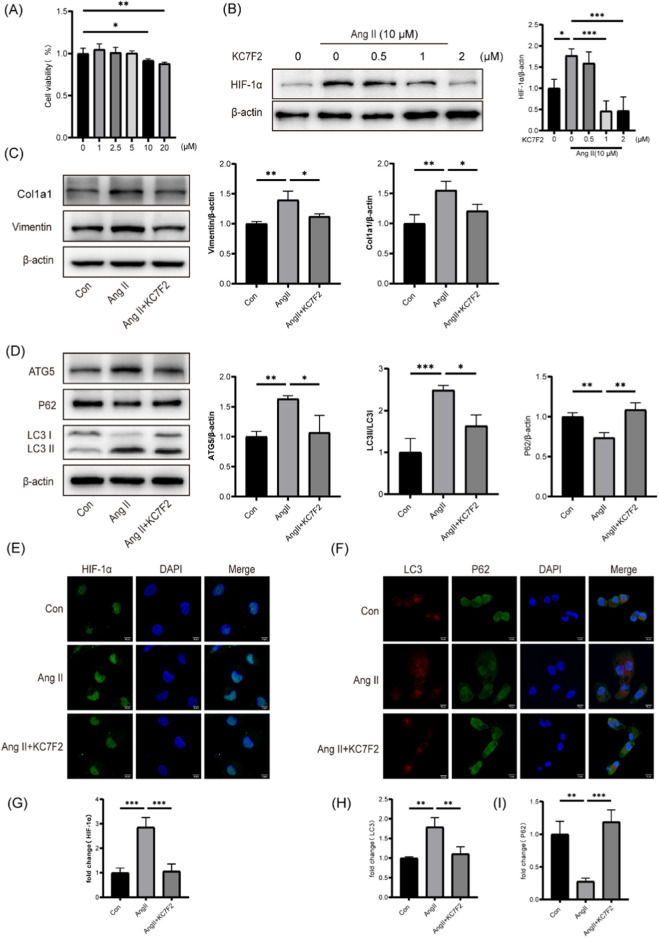
Inhibition of HIF-1α ameliorated Ang Ⅱ-induced autophagy and fibrosis in HK-2 cells **(A)** CCK-8 assay was employed to evaluate cell viability. **(B)** Protein level of HIF-1α in HK-2 cells **(C)** Protein levels of Col1a1 and Vimentin in HK-2 cells following HIF-1 inhibition. **(D)** Protein levels of ATG5、P62 and LC3B in HK-2 cells following HIF-1 inhibition **(E)** Immunofluorescence staining of HIF-1α in HK-2 cells. **(F)** Immunofluorescent staining for LC3 (shown in green) and p62 (shown in red). Scale bar, 10 μm **(G)** Quantitative analysis of HIF-1α fluorescence intensity. **(H)** Quantitative analysis of LC3 fluorescence intensity. **(I)** Quantitative analysis of P62 fluorescence intensity. *p < 0.05, **p < 0.01, ***p < 0.001.

To further investigate the effect of HIF-1α on hypertensive kidney injury, experiments were conducted on HK-2 cells. According to CCK8 cell viability results, a concentration of 10 μM Ang II was selected for the experiments (Figure 4A). Western blotting analysis revealed a significant reduction in the expression of HIF-1α protein in HK-2 cells when treated with one or 2 μM KC7F2 (Figure 4B). Therefore, a concentration of 1 μM KC7F2 was chosen for subsequent cell culture experiments. Upon exposure of HK-2 cells to Ang II, the expression levels of fibrosis markers, such as Col1a1 and Vimentin, were significantly elevated. However, after treatment with KC7F2, a reduction in the expression of these fibrosis markers was observed, as confirmed by Western blotting analysis (Figure 4C). Additionally, to determine the role of HIF-1α in autophagy regulation, we examined autophagy-related markers. Western blotting results showed an increase in LC3B-II and ATG5 expression, accompanied by a decrease in P62 expression following Ang II stimulation, indicating sustained autophagy activation in the cells (Figure 4D). Immunofluorescence results corroborated the Western blotting findings (Figures 4E–I).

### 3.8 Overexpression of HIF-1α exacerbated AngⅡ-induced fibrosis and autophagy in HK-2 cells

**FIGURE 5:**
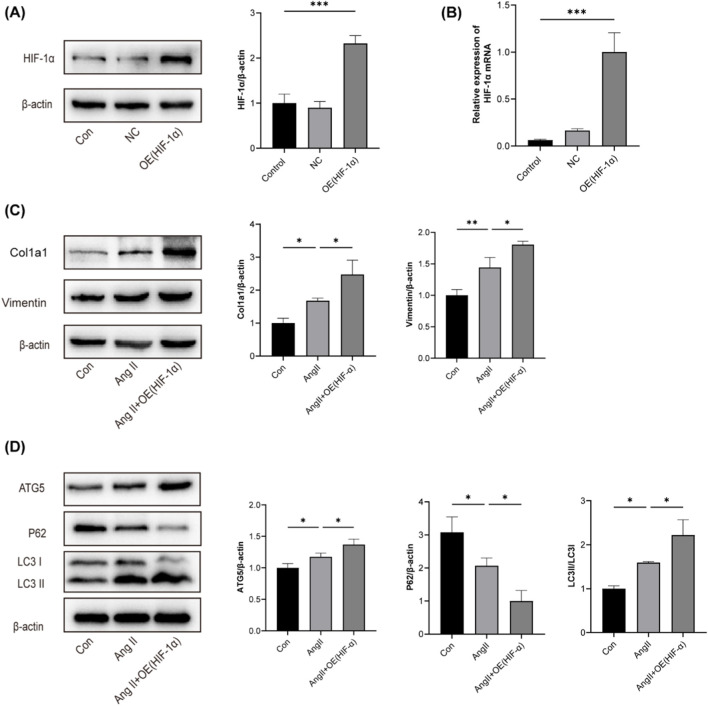
Overexpression of HIF-1α exacerbated Ang Ⅱ-induced fibrosis and autophagy in HK-2 cells **(A)** The effects of HIF-1α overexpression on HIF-1α protein levels in HK-2 cells were examined using Western blotting. **(B)** The effects of HIF-1α overexpression on HIF-1α mRNA levels in HK-2 cells were examined using RT-qPCR **(C)** Expression of Col1a1 and Vimentin in HK-2 cells was analyzed using Western blotting assay. **(D)** Expression of ATG5、P62 and LC3B in HK-2 cells was analyzed using Western blotting assay. *p < 0.05, **p < 0.01, ***p < 0.001.

To further study the role of HIF-1α in hypertensive renal fibrosis, a lentiviral vector overexpressing HIF-1α was constructed. Western blotting and RT-qPCR were used to analyze HIF-1α mRNA and protein expression (Figures 5A, B). HIF-1α overexpression exacerbated the expression of Col1a1, Vimentin, LC3B-II, and ATG5 induced by Ang II, and reduced the expression of P62 (Figures 5C, D). These results suggest that HIF-1α overexpression significantly enhances Ang II-induced tubular cell fibrosis and autophagic flux.

### 3.9 CQ, an autophagy inhibitor, mitigated ang II-induced fibrosis by inhibiting autophagy

**FIGURE 6:**
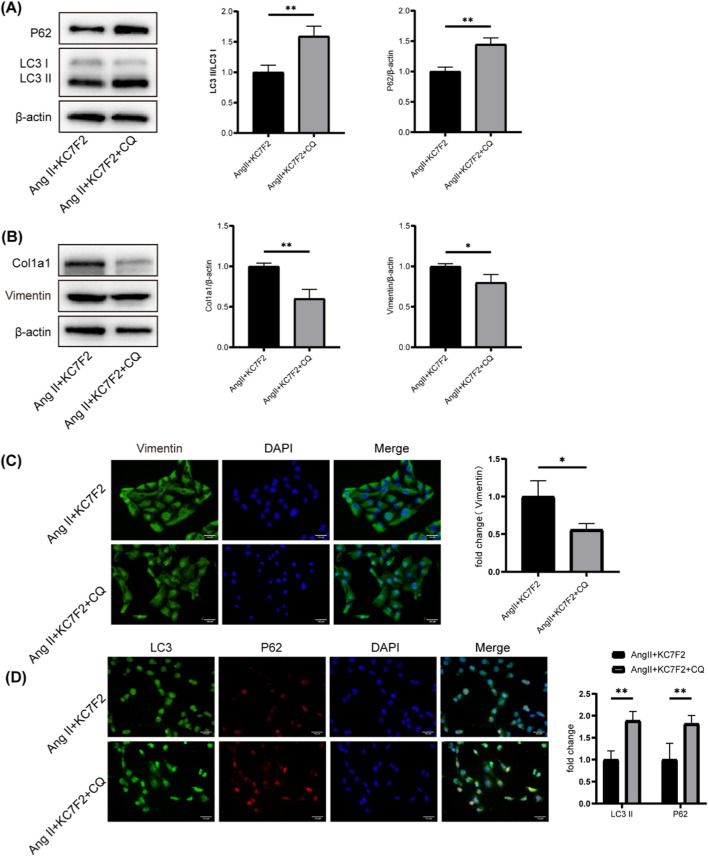
CQ, an autophagy inhibitor, mitigated Ang II-induced fibrosis by inhibiting autophagy **(A)** Western blotting analysis of p62 and LC3B in HK-2 cells treated with CQ. **(B)** Western blotting analysis of Col1a1 and Vimentin in HK-2 cells treated with CQ **(C)** The IF staining of Vimentin in HK-2 cells. **(D)** The IF staining of p62 and LC3 in HK-2 cells. *p < 0.05, **p < 0.01.

To further investigate the impact of autophagy on renal fibrosis progression, HK-2 cells were treated with the lysosomal inhibitor chloroquine (CQ) in conjunction with co-incubation of angiotensin II and KC7F2. We observed that the Ang II + KC7F2+CQ group exhibited increased expression of LC3B-II and p62 compared to the Ang II + KC7F2 group (Figure 6A). Additionally, we found a decrease in Col1a1 and Vimentin expression in the Ang II + KC7F2+CQ group compared to the Ang II + KC7F2 group (Figure 6B). This result was further validated by immunofluorescence (Figures 6C, D). These findings suggest that the HIF-1α inhibitor, KC7F2, can ameliorate Ang II-induced fibrosis in HK-2 cells by inhibiting autophagy.

## 4 Discussion

Physical exercise has been established as an effective non-pharmacological intervention for managing chronic kidney disease (CKD). It enhances kidney function and helps control key risk factors and progression of CKD, such as hypertension, hyperglycemia, and obesity (Liu et al., 2024a). Moderate exercise has been shown to reduce renal pathological changes and interstitial fibrosis caused by hypertension (Duan et al., 2021; Huang C. et al., 2018). Additionally, some studies suggest that HIIT may offer superior renal protection and blood pressure reduction compared to traditional aerobic exercise (Huang et al., 2018a; Tucker et al., 2015). However, contrary to expectations, only MICT produced beneficial outcomes for SHRs, while HIIT did not show significant protective effects. Our findings indicated that MICT had lowered blood pressure, enhanced kidney function, and alleviated pathological damage to the kidneys. Concurrently, Western blot analysis revealed that MICT significantly downregulated the expression of renal fibrosis-related proteins in SHR. In contrast, high-intensity interval training (HIIT) exhibited limited efficacy in modulating these fibrotic markers while demonstrating selective improvement in DBP. A previous meta-analysis demonstrated that high-intensity interval training (HIIT) is more effective than moderate-intensity continuous training (MICT) in reducing blood pressure, particularly in lowering diastolic blood pressure (DBP), which may be attributed to the superior efficacy of HIIT in improving arterial stiffness (Leal et al., 2020). Study has suggested that the reduction in DBP following HIIT may be associated with improvements in endothelial function and redox status (Dekleva et al., 2017). Additionally, the decrease in DBP during high-intensity exercise may result from enhanced myocardial contractility induced by vigorous physical activity (Coates et al., 2023). Specifically, HIIT has been shown to significantly improve left ventricular diastolic function compared to MICT, as evidenced by increased E/A ratio, accelerated early diastolic propagation velocity, and shortened untwisting time, all of which may contribute to the reduction in DBP (Huang et al., 2019a). However, HIIT did not demonstrate any improvement in renal function. One possible explanation is that the high exercise intensity in HIIT may induce a greater stress response in diseased rats, thereby counteracting the potential benefits of exercise. Research has shown that intense exercise makes the kidneys more susceptible to ischemic injury (Bongers et al., 2018). Furthermore, strenuous exercise has been demonstrated to reduce renal plasma flow and glomerular filtration rate, leading to significant proteinuria (Kodikara et al., 2023; Spada et al., 2018). Current evidence regarding the effects of HIIT on renal function remains inconsistent, likely due to differences in HIIT protocol designs. Our findings suggest that moderate-intensity exercise provides greater benefits in protecting renal function and reducing renal fibrosis, whereas HIIT is ineffective in reversing hypertension-induced kidney damage.

HIF-1α expression was reported to be significantly elevated in the kidneys of SHRs compared to WKY rats, suggesting its pivotal role in HN (Qian et al., 2021). Multiple studies have explored HIF-1α′s involvement in tubulointerstitial fibrosis. For instance, findings have shown that silencing HIF-1α in the renal epithelium suppresses tubulointerstitial fibrosis in rats with unilateral ureteral obstruction, whereas overexpression of HIF-1α in renal tubular epithelial cells promotes fibrosis (Liu et al., 2024b). Additionally, increased HIF-1α levels were found to exacerbate renal damage in both HN models and cisplatin-induced chronic kidney disease (Luo et al., 2015; Zhao et al., 2021). These studies suggest that HIF-1α activation plays a role in promoting renal fibrosis. In our study, we evaluated the impact of MICT and HIIT on HIF-1α expression and fibrosis in SHR kidneys. The results demonstrated that MICT reduced both HIF-1α levels and fibrosis, whereas HIIT had no significant effect on the kidneys. The observed reduction in renal fibrosis following MICT intervention appears to be associated with decreased HIF-1α expression in SHRs.

HIF-1α, a key regulator of hypoxia, may contribute to the development of renal fibrosis by activating autophagy in the kidney (Liu et al., 2024b). Renal tubular epithelial cells, due to their high metabolic demands and low oxygen partial pressure, are particularly vulnerable to hypoxia-induced damage (Livingston et al., 2023). Autophagy, which maintains cellular homeostasis by degrading cytoplasmic components, can be triggered by metabolic, genotoxic, or hypoxic conditions and serves as an adaptive mechanism for cell survival (Liang et al., 2022). Activation of autophagy in proximal tubular cells has been shown to promote renal fibrosis in models of UUO and acute kidney injury (Liu et al., 2024b; Livingston et al., 2023). In animal experiments, AKT/mTOR signaling pathway-related proteins were found to be inhibited in the kidneys of SHRs, leading to enhanced autophagy. Given the central roles of HIF-1α and autophagy in renal fibrosis, these mechanisms were further investigated *in vitro*. HK-2 cells stimulated with Ang II were used to build a hypertensive nephropathy model. Our study demonstrated that Ang II induced HIF-1α expression in HK-2 cells. The specific HIF-1α inhibitor, KC7F2, significantly blocked autophagy and fibrosis in HK-2 cells, while overexpression of HIF-1α exacerbated both. HIF-1α has been shown to worsen renal fibrosis by promoting autophagy (Liu et al., 2024a).

However,the role of autophagy in Ang II-induced renal fibrosis is unclear. Our findings have demonstrated that KC7F2, a HIF-1α inhibitor, reduced Ang II-induced Col1a1 and Vimentin upregulation in HK-2 cells. Hence, we co-treated Ang II-stimulated HK-2 cells with CQ and KC7F2. In Ang II-exposed HK-2 cells, CQ further inhibited the autophagic flux and mitigated Col1a1 and Vimentin protein levels. These results suggested HIF-1α inhibition may reduce Ang II-induced renal fibrosis by decreasing autophagy.

## 5 Conclusion

In conclusion, our study suggests that MICT appears to be more effective in improving renal function and reducing fibrosis in SHRs, compared to HIIT. This effect may be mediated by inhibiting HIF-1α in renal tubular epithelial cells, thereby reducing excessive autophagy.

## Data Availability

The raw data supporting the conclusions of this article will be made available by the authors, without undue reservation.
