# Supplementary material for: Moderate intensity continuous training mitigates hypertension-induced renal fibrosis by inhibiting HIF-1α-mediated autophagy
Source: Front Physiol. 2025 Mar 27;16:1529811. doi: 10.3389/fphys.2025.1529811 (PMC11983329; doi:10.3389/fphys.2025.1529811)
Supplement: Supplementary file 1 [file Table1.docx]

**Supplemental Information**

**Table 1.** exercise protocol.

| SHR-M group | | | | SHR-H group | | | | |
| --- | --- | --- | --- | --- | --- | --- | --- | --- |
| Week | Speed  (m/s) | Time  （min） | Distance  （km） | Speed  (m/s) | Time(min) | | Number of cycles | Distance (km) |
|  |  |  |  |  | Sprint time | Interval time |  |  |
| 1 | 8-10 | 60 | 2.4-3.0 | 14-16 | 1 | 2 | 10 | 4.8-5.1 |
| 2 | 12-14 | 60 | 3.6-4.2 | 21-24 | 1 | 2 | 10 | 3.6-4.0 |
| 3 | 16-18 | 60 | 4.8-5.4 | 28-30 | 1 | 2 | 10 | 4.8-5.1 |
| 4 | 16-18 | 60 | 4.8-5.4 | 28-30 | 1 | 2 | 10 | 4.8-5.1 |
| 5 | 16-18 | 60 | 4.8-5.4 | 28-30 | 1 | 2 | 10 | 4.8-5.1 |
| 6 | 16-18 | 60 | 4.8-5.4 | 28-30 | 1 | 2 | 10 | 4.8-5.1 |
| 7 | 16-18 | 60 | 4.8-5.4 | 28-30 | 1 | 2 | 10 | 4.8-5.1 |
| 8 | 16-18 | 60 | 4.8-5.4 | 28-30 | 1 | 2 | 10 | 4.8-5.1 |
| 9 | 16-18 | 60 | 4.8-5.4 | 28-30 | 1 | 2 | 10 | 4.8-5.1 |
| 10 | 16-18 | 60 | 4.8-5.4 | 28-30 | 1 | 2 | 10 | 4.8-5.1 |
